# Single-cell transcriptomics reveals the drivers and therapeutic targets of lymph node metastasis in lung adenocarcinoma

**DOI:** 10.18632/aging.204890

**Published:** 2023-07-22

**Authors:** Xin Ji, Zihao Wang, Guige Wang, Lijun Tang, Zhijun Han

**Affiliations:** 1 Department of Nuclear Medicine, The First Affiliated Hospital of Nanjing Medical University, Jiangsu Province Hospital, Nanjing, China; 2 Department of Breast Surgery, Peking Union Medical College Hospital, Chinese Academy of Medical Sciences and Peking Union Medical College, Beijing, China; 3 Department of Thoracic Surgery, Peking Union Medical College Hospital, Chinese Academy of Medical Sciences and Peking Union Medical College, Beijing, China

**Keywords:** lymph node metastasis, lung adenocarcinoma, immune microenvironment, drug sensitivity

## Abstract

Lymph node metastasis (LNM) is usually the most common metastatic pathway in lung adenocarcinoma (LUAD) and is associated with a poorer prognosis and higher possibility of recurrence. Therefore, discovering the drivers and therapeutic targets of LNM is important for early and non-invasive detection of patients with a high risk of LNM and guiding individualized therapy. Various cell constitutions of the primary tumor and lymph node microenvironment was characterized based on scRNA-seq data. The copy number variation (CNV) analysis was performed to probe clonal structures and origins of metastatic lymph nodes, and found 6q loss and 20q gain may drive LNM in LUAD. Then a LNM-related cell subset, named Scissor+ cells, was identified using the Scissor algorithm. And cell-cell communication network among Scissor+ cells and microenvironment was further analyzed. Besides, a pro-LNM signature was subsequently constructed based on 27 genes using pseudotime trajectory analysis and gene set variation analysis. The pro-LNM signature showed a significant correlation with *N* stage and a good predictive ability of LUAD survival. At last, we identified that erastin and gefitinib could potentially inhibit LNM by targeting Scissor+ cells based on the drug sensitivity data of the cancer cell lines, which provided new insights for LUAD therapy.

## INTRODUCTION

Lung adenocarcinoma (LUAD) is the most prevalent lung cancer histologic subtype and accounts for almost half of all lung cancers [1, 2]. Lymph node metastasis (LNM) is generally considered to be one of the most common metastatic pathways of LUAD and is associated with a poorer prognosis and higher possibility of locoregional or distant recurrence [3]. Patients with LNM usually require more extensive systemic treatment. Therefore, the identification of drivers of LNM may be important for early identification of patients with unfavorable prognosis and guiding individualized treatment, thus further reducing overall mortality.

Relevant studies on the prediction of LNM in lung cancer are limited, and most are focused on clinical factors, serum biomarkers and radiomics signature, while effective predictors remain lack of investigations [3–5]. Several genes were also identified as the LNM indicators based on transcriptome profile [6, 7]. However, since metastasis is a multi-gene process and involves the interaction between tumor and the surrounding microenvironment, these single-gene predictors may not provide a complete picture of the mechanism underlying lymph node metastasis.

With the advancement of single-cell RNA-sequencing (scRNA-seq) technology, cellular and molecular features associated with tumor progression and microenvironment have been increasingly investigated [8, 9]. Previous scRNA-seq studies related to LUAD have focused on molecular features of tumorigenesis and progression [10–12]. In the present study, the clonal origin and transcriptional trajectory of LUAD were analyzed to reveal the genetic drivers for LNM in LUAD. Besides, a pro-LNM signature and several drug candidates were identified, which may facilitate early prediction of LNM and provide new insights into cancer therapy.

## RESULTS

### scRNA landscape of the tumor microenvironment in LUAD primary tumor (PT) and lymph node (LN) samples

**Figure 1:**
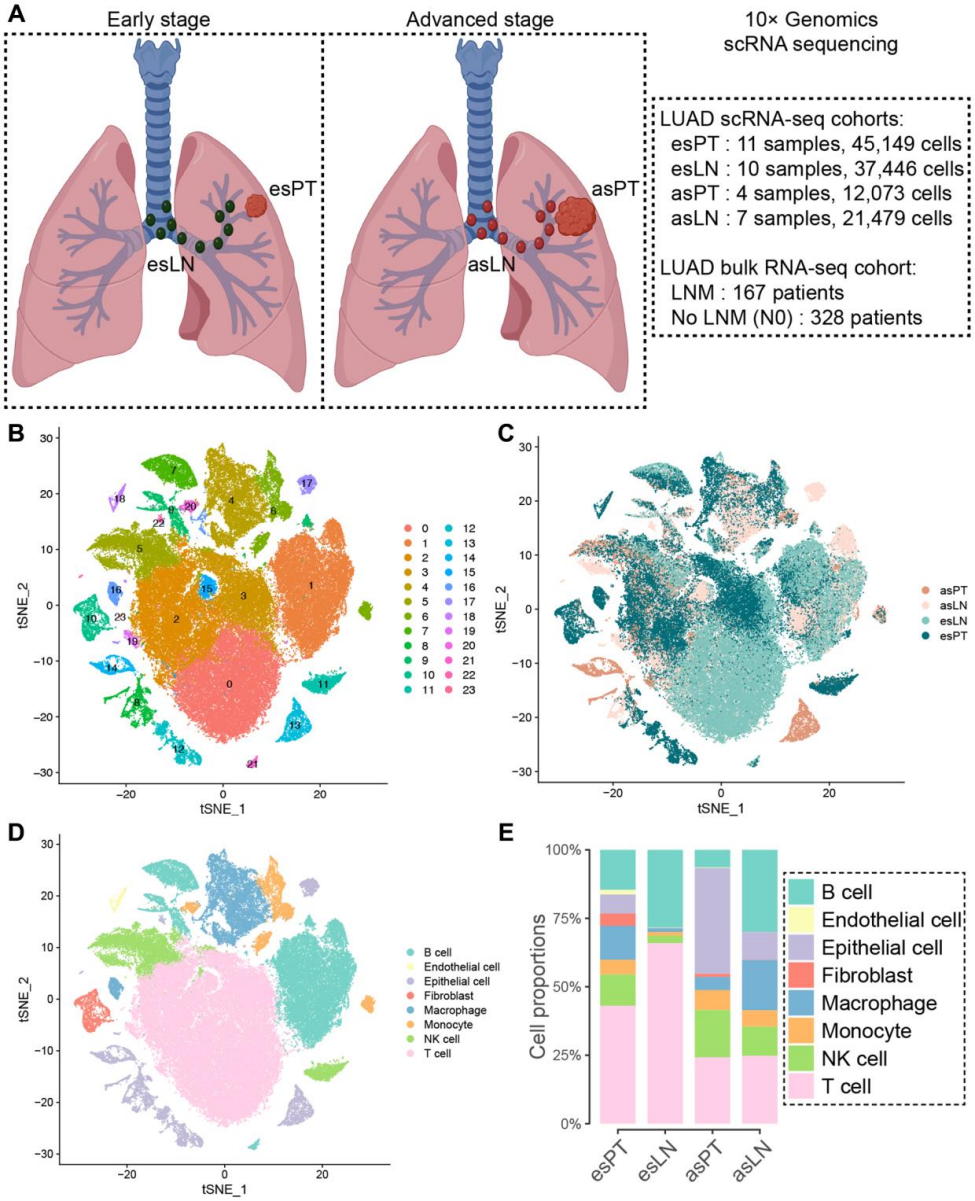
**Clustering of cells in tumor and lymph node samples from early and advanced stage LUAD patients.** (**A**) Overview of the scRNA-seq and bulk RNA-seq cohorts. (**B**) T-SNE visualization of 24 cell clusters from scRNA-seq cohort samples, and (**C**) showing various origin of the cells. (**D**) Eight cell clusters were manually annotated based on DEGs according to canonical cell lineage markers. (**E**) Comparison of the cell constitution among different tissues shown by the bar plot. Abbreviations: LUAD: lung adenocarcinoma; esPT: primary tumor in early stage LUAD; esLN: lymph node in early stage LUAD; asPT: primary tumor in advanced stage LUAD; asLN: lymph node in advanced stage LUAD; LNM: lymph node metastasis.

To investigate the heterogeneity of cell phenotype between primary tumors and metastasis lymph nodes in LUAD, we included the data of 11 primary tumor (esPT, 45,149 cells) and 10 normal lymph node (esLN, 37,446 cells) samples from early stage LUAD patients, as well as four primary tumor (asPT, 12,073 cells) and seven metastatic lymph node (asLN, 21,479) tissues from advanced stage LUAD patients (Figure 1A). After data processing and quality control, cells from PT and LN samples were automatically clustered into 24 subgroups using principle component analysis (PCA) and t-distributed stochastic neighbor embedding (t-SNE) (Figure 1B, 1C). Then cell clusters were annotated based on the DEGs according to canonical cell lineage markers (Figure 1D). Since LUAD originates from the bronchial mucosal epithelium, we found that the proportion of epithelial cells (ECs) increased significantly in asPT and asLN tissues, with 38.6% and 10.0%, respectively. ECs only accounted for 6.9% in esPT and were absence in esLN. Immune cells were the predominant proportion in esPT (86.9%) and esLN (99.8%), especially T cells, which accounted for 43.1% of total cells in esPT and 65.9% in esLN (Figure 1E). However, the proportions of T cells declined significantly in asPT and asLN, with a declination of 18.9% and 41.1%, respectively. B lymphocytes also decreased in asPT compared with esPT but increased in asLN (30.0%) compared with esLN (28.4%), although with no significance. Besides, macrophages largely increased in asLN (18.5%) when compared to asPT (4.8%) and esLN (1.3%).

### Clonal evolution and hallmark signature of malignant ECs (maECs)

**Figure 2:**
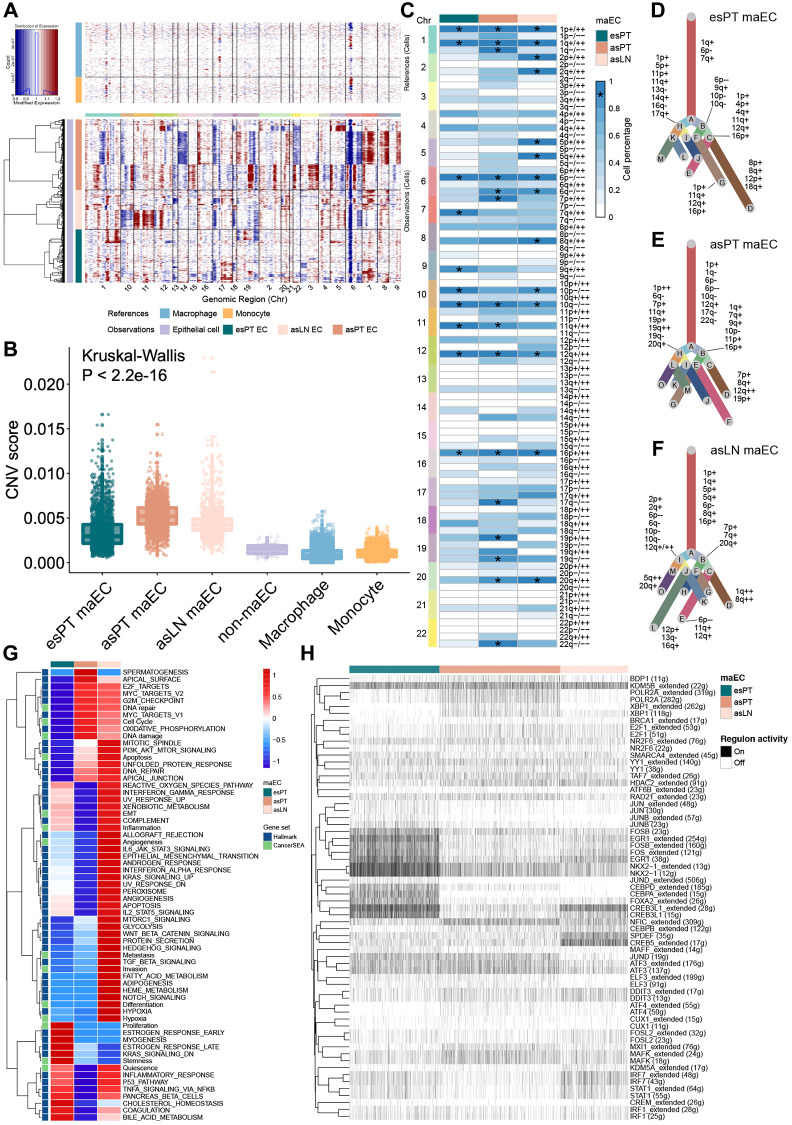
**Copy number variation analysis of epithelial cells from primary tumor and metastasis lymph node.** (**A**) Representative hierarchical heatmap showing large-scale CNVs in maECs from various samples. Gains (red) or losses (blue) were inferred by averaging the expression over 100 gene stretches on the respective chromosomes. (**B**) The CNV score facilitated quantitatively determining malignant cells. (**C**) The summary plot of the CNV profile of malignant ECs from esPT, asPT and asLN. CNVs were converted to the chromosome arm level change and simplified as gain or loss. (**D**–**F**) The clonal evolutionary trees of maECs from esPT, asPT and asLN, respectively. The length of branch is determined by the percentage of cells containing the corresponding CNV in subclones. (**G**) The heatmap of GSVA demonstrated differences in enriched pathways among maECs from esPT, asPT and asLN. (**H**) The hierarchical clustering heatmap of enriched transcription regulon using SCENIC analysis. ^*^, means presenting in >90% of tumor cells. Abbreviations: CNVs: copy number variations; maECs: malignant epithelial cells; esPT: primary tumor in early stage LUAD; esLN: lymph node in early stage LUAD; asPT: primary tumor in advanced stage LUAD; asLN: lymph node in advanced stage LUAD; GSVA: gene set variation analysis; SCEINC: single-cell regulatory network inference and clustering.

To probe the clonal structure and origins of malignant cells, inferCNV algorithm was applied to analyze the CNV and clonality of the ECs from esPT, asPT and asLN (Figure 2A). Compared with the reference cells, 2,303 ECs from esPT, 3,093 cells from asPT and 1,731 cells from asLN were classified as malignant cells due to high CNV scores, the last 88 ECs with low CNV scores and the reference cells were considered non-malignant cells (Figure 2B). The 1p gain, 1q gain, 6p loss, 10q loss, 12q gain and 16p gain appeared in more than 80% maECs from esPT, asPT and asLN (Figure 2C). And the gain of 7p, 19p, 20q and loss of 1q, 17q, 19q, 22q were observed in asPT maECs compared with esPT. And the gain of 2p, 2q, 5p, 5q, 8q and loss of 6q, 10p were observed in asLN compared with asPT. Interestingly, the 6q loss and 20q gain existed in most maECs from asPT and asLN rather than esPT, which might be the driver of lymph node metastasis of LUAD. A clonal evolutionary tree was then plotted based on the subclone CNV results (Figure 2D–2F). Multiple CNVs in subclones were observed in each sample, which revealed the previously unappreciated heterogeneity and complexity of CNV in epithelia cells from LUAD primary tumors and lymph nodes.

The gene set variation analysis (GSVA) analysis of maECs revealed relatively high activation level of E2F target, MYC target, DNA repair and G2M checkpoint pathways in both asPT and asLN. The cell cycle and DNA damage pathway were upregulated in asPT, while asLN exhibited increased activation of PI3K-AKT-mTOR, IL2-STAT5, IL6-JAK-STAT3, IFNα, TNFβ, KRAS, EMT, NOTCH, angiogenesis, metastasis, invasion signaling (Figure 2G). To illustrate the transcriptional state across ECs in LUAD, potential co-expression modules and their cis-regulatory motifs were identified using single-cell regulatory network inference and clustering (SCENIC) analysis (Figure 2H). Histone demethylase KDM5B was found to be actively expressed in all subclusters of maECs, which could promote tumor invasion and induce immune evasion through cytosolic RNA or DNA sensing pathways and subsequent interferon-1 responses [13]. The most over-represented elements included NKX2, EGR1 and FOS were enriched in ECs from esPT. While NFIC, XBP1, MAFK were relatively high expressed in asPT maECs. And for asLN maECs, SPDEF and CREB5 were highly expressed.

### Pseudotime trajectory identified LNM-dependent gene patterns

**Figure 3:**
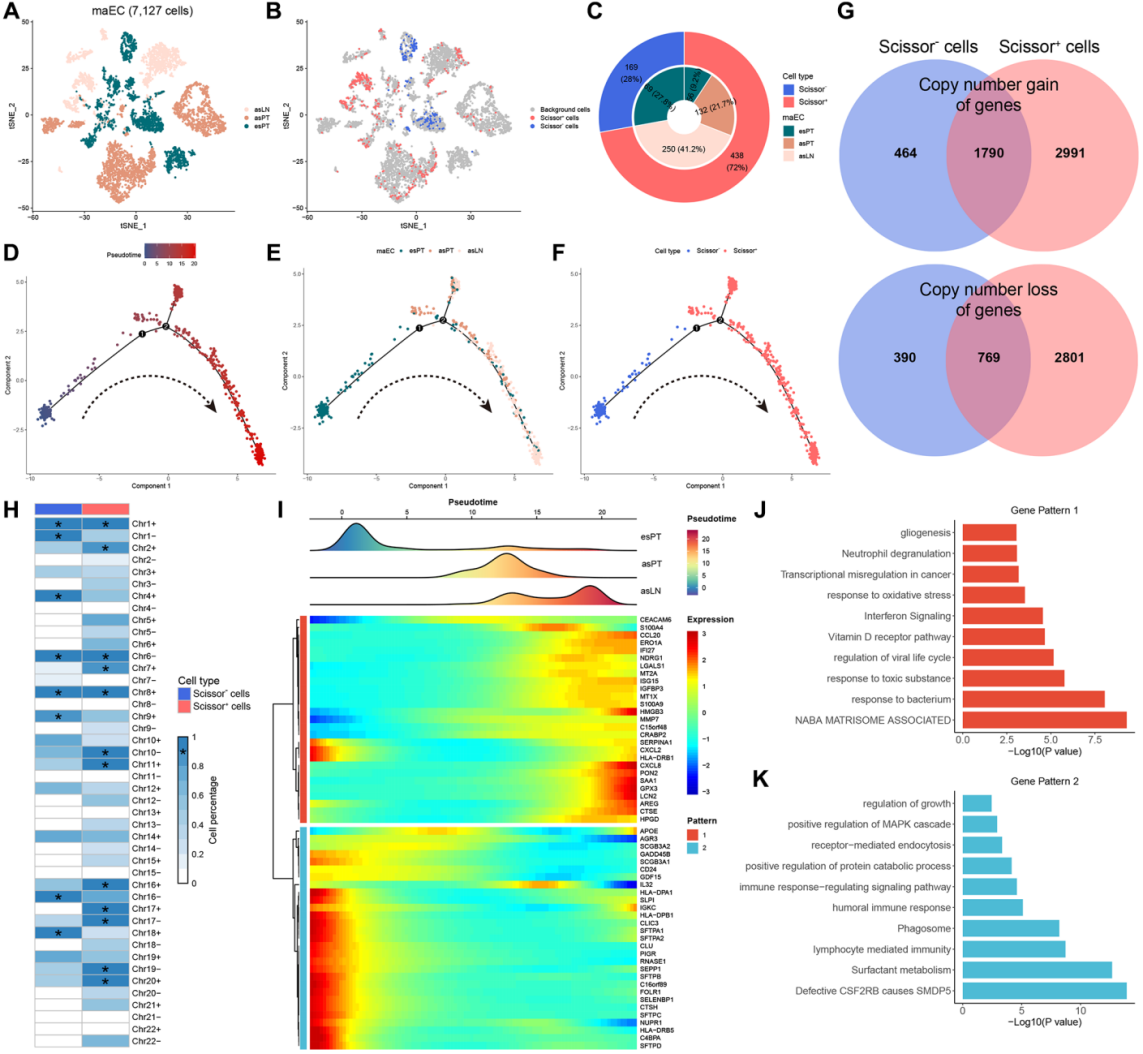
**Trajectory analysis of malignant ECs.** (**A**, **B**) T-SNE plots of 7127 malignant ECs color-coded by origins and scissor clusters, respectively. (**C**) The pie chart showing the constitution of cells with various origins and the distribution of scissor subclusters. (**D**–**F**) The evolutionary phylogenetic trees of Scissor^+^ and Scissor^−^ cells annotated by pseudotime, cell origins and cell types, respectively. (**G**) A Venn diagram for summarizing gene variation between Scissor^+^ and Scissor^−^ cells. Blue represents Scissor^−^ cells and red represents Scissor^+^ cells. (**H**) The summary plot of the CNV profile of Scissor^+^ and Scissor^−^ cells. CNVs were converted to the chromosome arm level change and simplified as gain or loss. (**I**) Heatmap of 56 branch-dependent genes identified by BEAM. (**J**, **K**) The enriched hallmark pathways of two gene patterns by GSVA. ^*^, means presenting in >90% of tumor cells. Abbreviations: ECs: epithelial cells; T-SNE: t-distributed stochastic neighbor embedding; CNV: copy number variation; BEAM: branch expression analysis modeling; GSVA: gene set variation analysis.

Under the guidance of phenotypic information from bulk RNA sequencing data, Scissor algorithm was applied to identify the cells most related to LNM from single-cell data. All 7,127 malignant cells were included for dimensional reduction and clustering (Figure 3A). Then, 438 cells were determined as Scissor^+^ cells, which showed a positive correlation with LNM phenotype, and 169 cells were Scissor^−^ cells which were negatively correlated with LNM phenotype, while the last 6,520 cells were regarded as background cells (Figure 3B). Among Scissor^+^ cells, cells from esPT, asPT and asLN accounted for 9.2%, 21.7% and 41.2% of total cells, respectively. While Scissor^−^ cells were all from esPT (Figure 3C). Based on pseudotime ordering, Scissor^+^ and Scissor^−^ cells were projected onto different branches using trajectory analysis (Figure 3D–3F). All Scissor^−^ cells presented at the initial stage of differentiation that all belonged to esPT, while Scissor^+^ cells enriched in the middle and terminal stage of differentiation and finally divided into two branches. The two branches were mostly enriched in asPT cells and asLN cells, respectively. Venn diagram indicated that Scissor^+^ cells existed 2991 newly gain of genes and 2801 loss of genes when compared to Scissor^−^ cells (Figure 3G). Previously identified potential drivers of LNM, namely the 6q loss and 20q gain, were also found in Scissor^+^ cells (Figure 3H).

BEAM analysis was performed to identify branch-dependent genes across branches. A total of 56 genes involved in the regulation of cell differentiation were identified, which were further divided into two gene patterns (Figure 3I). The 27 genes in gene pattern 1 were named as pro-LNM signature, as their expression was thought to be associated with LNM. Among them, genes related to cell proliferation (HMGB3, GPX3, SAA1), ferroptosis (C15orf48, LCN2), immune microenvironment (CCL20, CXCL8) and epithelial-to-mesenchymal transition (AREG) were gradually upregulated alongside the trajectory differentiation process. Afterwards, GSVA revealed that transcriptional misregulation in cancer, response to oxidative stress, interferon signaling and matrisome-associated pathways were significantly enriched in gene pattern 1 (Figure 3J). While gene pattern 2 demonstrated quite different enriched pathways, including positive regulation of MAPK cascade, immune response-regulating signaling pathways, lymphocyte-mediated immunity, etc., (Figure 3K).

### Intercellular communication of Scissor^+^ cells in tumor microenvironment

**Figure 4:**
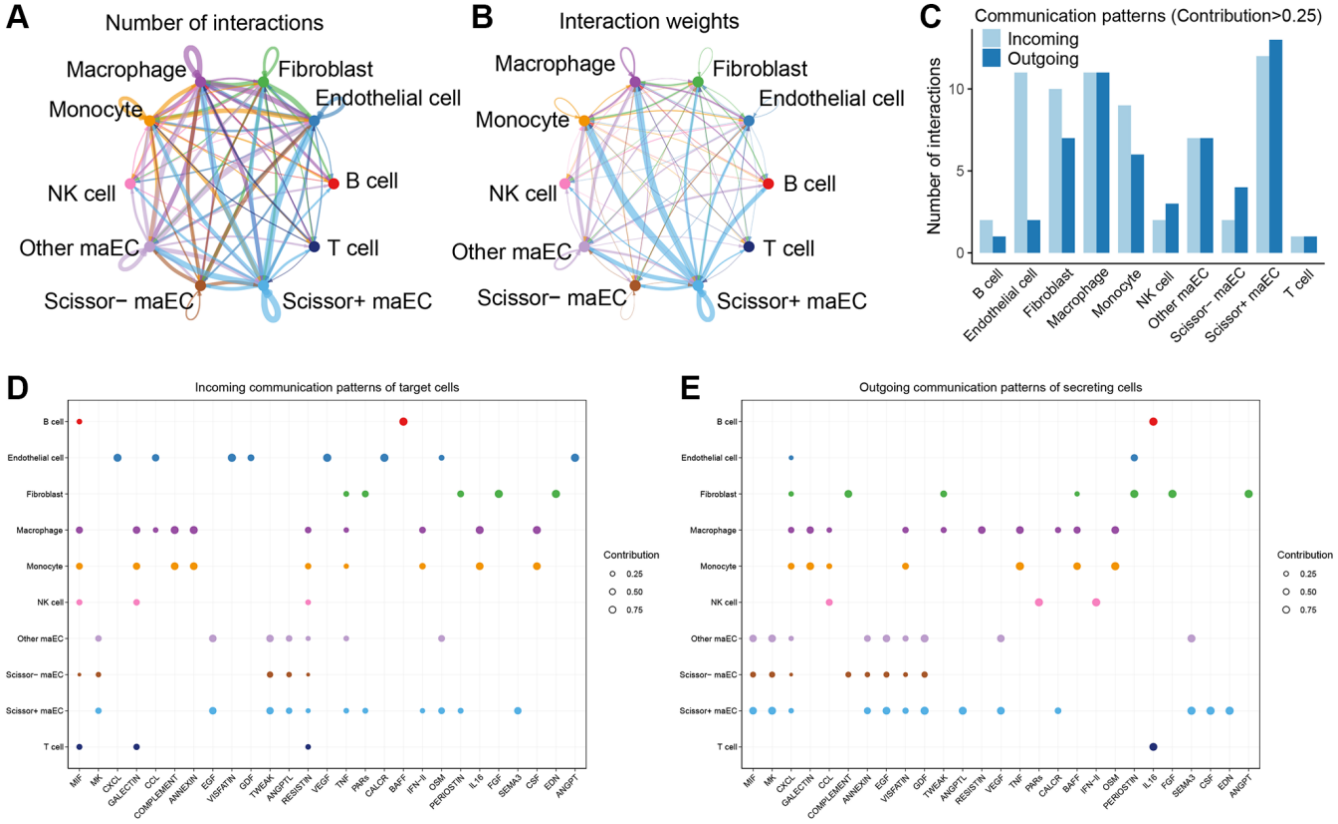
**Cell-cell communication network in tumor microenvironment.** (**A**, **B**) Circle plots showing the number (left) and strength (right) of cell interactions in tumor microenvironment. The edge width is proportional to the numbers or strength of significant ligand-receptor pairs. (**C**) Comparisons of the numbers of intercellular interactions with contribution degree more than 0.25 among all cell clusters. (**D**) Dot plots depict the incoming patterns of target cells, which shows the correspondence between the inferred latent patterns and cell clusters, as well as signaling pathways. (**E**) Outgoing communication patterns of secreting cells.

Tumor microenvironment plays a vital role in tumor progression and metastasis. To further investigate the intercellular communication network in the microenvironment, CellChat algorithm was applied to infer the ligand-receptor interactions among malignant epithelial cells and other cell clusters. As shown in Figure 4A, Scissor^+^ maECs were relatively active in the cell–cell interaction network, composing the biggest part of both incoming and outcoming communication with other cells. And the strength of interaction between Scissor^+^ maECs and immune cells like monocytes and macrophage were relatively strong, indicating the crucial role of Scissor^+^ maECs in tumor microenvironment (Figure 4B). Also, communication patterns indicated that Scissor^+^ maECs and macrophages play important role in tumor microenvironment (Figure 4C). According to the incoming communication patterns of the target cells, Scissor^+^ maECs dominantly responded to MK, EGF, TWEAK and SEMA3 signaling pathways (Figure 4D). And Scissor^+^ maECs drove MIF, MK, EGF, GDF, ANGPTL, VEGF, SEMA3, CSF, EDN signaling pathways as inferred by the outgoing communication patterns of the secreting cells (Figure 4E).

### The pro-LNM signature correlated with the LNM and prognosis of LUAD

**Figure 5:**
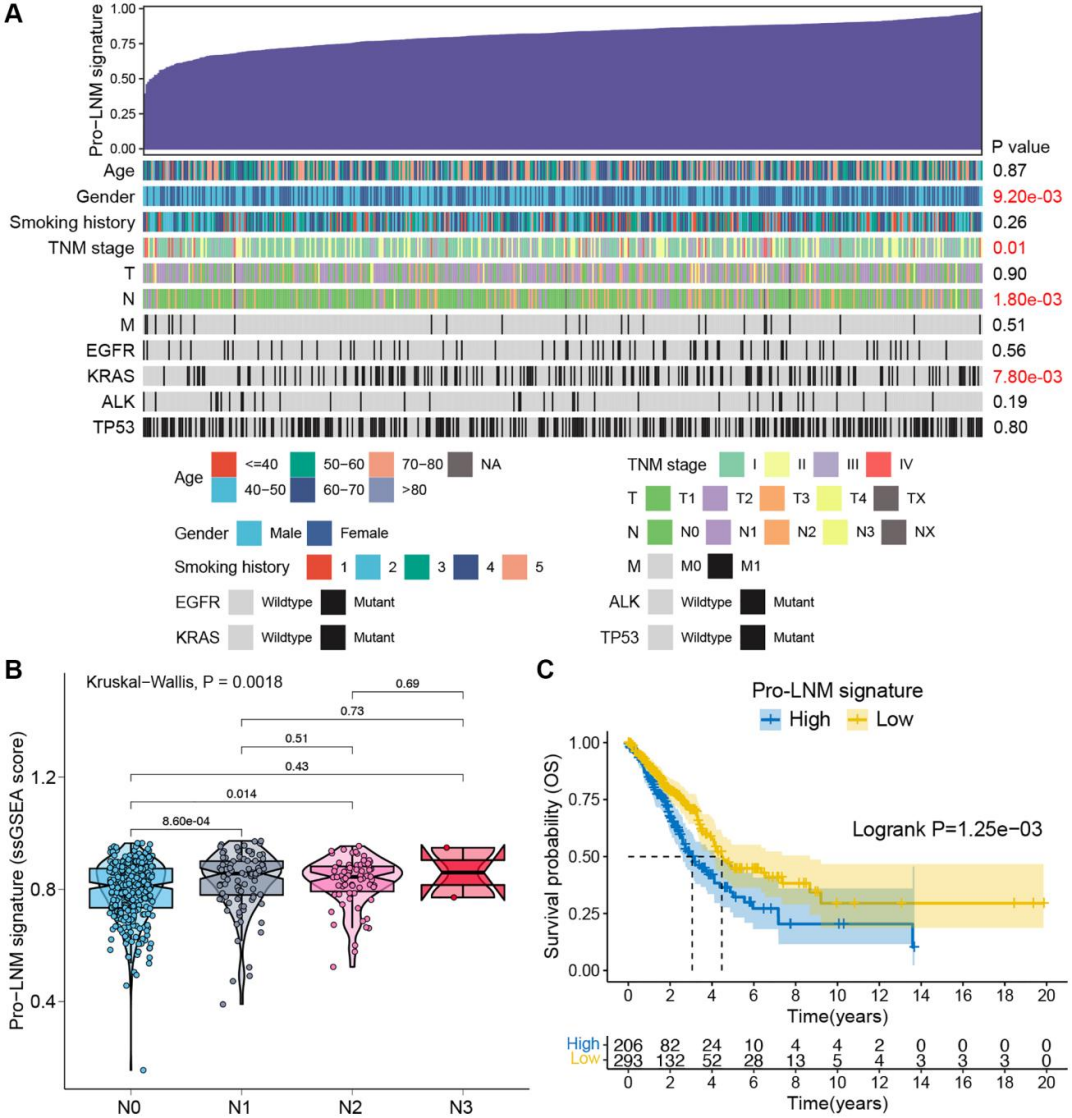
**Validation of pro-LNM signature associated with LNM and prognosis in the TCGA LUAD cohort.** (**A**) An overview of the association between the pro-LNM signature and clinicopathological parameters of LUAD patients. Columns showed patients ranked by pro-LNM signature from low to high (top row), and other rows represented the clinicopathological features. (**B**) Associations between pro-LNM signature and N staging in the TCGA LUAD cohort. (**C**) K-M survival analysis revealed significantly poorer OS in patients with high levels of pro-LNM signature. Abbreviations: LNM: lymph node metastasis; TCGA: The Cancer Genome Atlas; LUAD: lung adenocarcinoma; OS: overall survival.

To further investigate the relationship between pro-LNM signature and clinicopathological parameters, single sample gene set enrichment analysis (ssGSEA) was performed to calculate the pro-LNM signature ssGSEA score of each patient in the TCGA LUAD cohort. More advanced *N* stage was associated with higher ssGSEA scores (*p* = 0.0018). Gender, TNM stage, KRAS mutation status were also found to correlate with pro-LNM signature (Figure 5A). The Kruskal-Wallis test was performed to further analyze the association between *N* stage and pro-LNM signature. The ssGSEA scores were significantly higher in stages N1 and N2 than stage N0, with *p* values of 0.0009 and 0.014, respectively. While there were no significant differences among stage N1 to N3 (Figure 5B). Also, K-M survival analysis revealed that patients with a higher level of pro-LNM signature showed significantly poorer overall survival (*p* = 0.0013) (Figure 5C).

### Identification of the potential drugs targeting LNM

**Figure 6:**
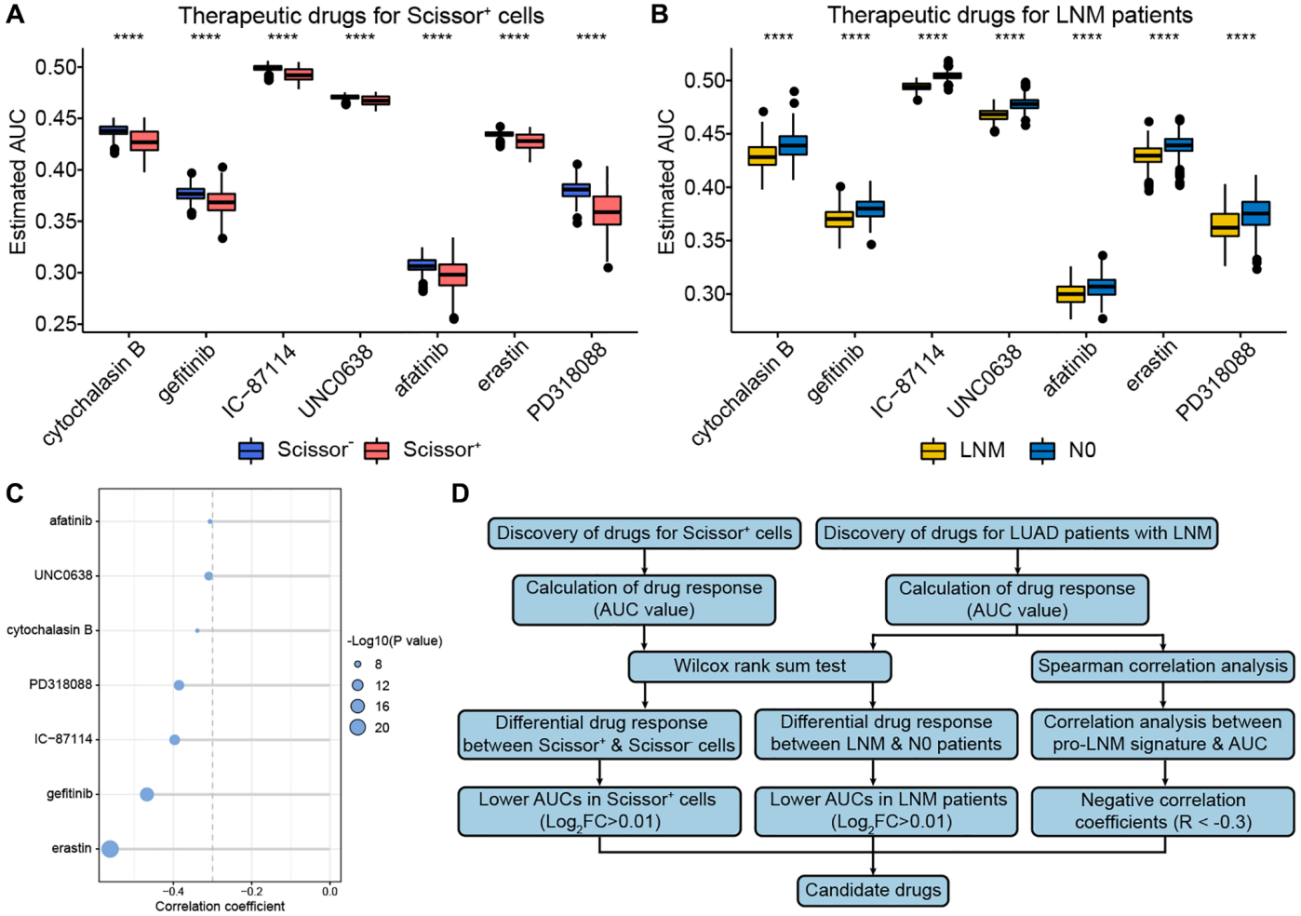
**Identification of potential drug targets for patients with LNM.** (**A**, **B**) The boxplots of differential drug response analysis in Scissor+ cells and LNM patients. (**C**) The Spearman’s correlation analysis between pro-LNM signature and AUC. (**D**) The overall workflow for identifying potential drug targets for patients with LNM. ^****^, means *p* < 0.001. Abbreviations: LNM: lymph node metastasis; AUC: area under the curve.

The pRRophetic algorithm was applied to identify the candidate drugs that inhibit LUAD LNM based on the drug sensitivity data of 46 LUAD cell lines in the CTRP database. We identified 74 compounds that performed higher sensitivity targeting Scissor^+^ cells and 15 compounds for patients with LNM in the LUAD cohort. By taking the intersections, seven drugs (erastin, gefitinib, IC-87114, PD318088, cytochalasin B, UNC06638 and afatinib) were finally identified to be the candidate drugs for the treatment of LUAD LNM. Besides, these seven drugs performed significant lower AUC values in Scissor^+^ cells and LNM patients and showed negative correlations with the pro-LNM signature (Figure 6A–6C). And the overall workflow was shown as Figure 6D. Further studies are required to validate the therapeutic value of these drugs in inhibiting LUAD lymph node metastasis.

## DISCUSSION

Lymph nodes are generally the first involved sites in the metastatic process of lung cancer, which is largely associated with a suboptimal prognosis. LUAD showed a higher propensity for LNM than squamous carcinoma and occurred LNM even in early stage [14, 15]. Besides, LUAD with LNM usually requires more extensive systemic treatment. Therefore, identification of LNM drivers is of great importance for prognosis prediction and treatment decision.

Tumor microenvironment plays a crucial role in tumorigenesis, metastasis and angiogenesis of LUAD [16]. And the infiltration of various immune cells was related to prognosis [17]. Consistent with previous studies [18, 19] we found that T and B lymphocytes dominated the early LUAD immune microenvironment, while both cell types reduced in advanced stage, suggesting an immunosuppressive environment, which may explain the immune escape in advanced stage LUAD. Besides, macrophages and monocytes increased in asLN compared to esLN, and cell-cell communication network analysis suggested their strong interactions with Scissor^+^ maECs, indicating that monocytes and macrophages may participate in the metastatic tumor cell growth in lymph nodes. Larionova et al. also confirmed that tumor associated macrophages and monocytes could upregulate CRYAB expression on tumor cells and activate the ERK1/2/Fra-1/ SLUG signaling pathway, which is associated with LNM [20].

Previous LNM predictors such as plasma CEA level, tumor size, pleural involvement and pathologic features had limited values in clinical work due to lack of accuracy or efficiency or invasive biopsies [21–23]. Therefore, effective biomarkers for predicting LNM are yet to be discovered. As metastasis is a multi-gene process and involves the interaction between tumor and the surrounding microenvironment, single-gene or single-pathway predictors such as LINE-1 [12] may not provide a complete picture of LNM. This study first identified a 27-gene pro-LNM signature based on hallmark genes that determine the trajectory differentiation. The signature included genes related to cell proliferation and migration (ERO1A, HMGB3, GPX3, SAA1), ferroptosis (C15orf48, LCN2), immune microenvironment (CCL20, CXCL8), epithelial-to-mesenchymal transition (AREG) and others. Besides, TCGA LUAD cohort were further included for validation, and the pro-LNM signature showed a significant correlation with *N* stage and a good predictive ability of LUAD survival, suggesting that it could be a promising biomarker for predicting LUAD LNM.

In addition, this study identified seven candidate drugs for LUAD patients with LNM. Among them, erastin and gefitinib showed superior properties in AUC and correlation analysis. Erastin is a classic ferroptosis inducer, which has shown promising pharmacological efficacy in cancer treatment. It has been confirmed that erastin could increase the sensitivity of non-small-cell lung cancer (NSCLC) cells to cisplatin and other chemotherapy and enhance the anti-tumor effect of radiotherapy in human-derived LUAD model [24–26]. Also, studies revealed that LCN2, a ferroptosis related protein, could promote tumor proliferation and adhesion through ferroptosis [27, 28]. In this study, LNM-associated malignant ECs were enriched with LCN2 and showed higher sensitivity to erastin, suggesting erastin could be a promising therapeutic option for LUAD patients with LNM. Gefitinib is an EGFR tyrosine kinase inhibitor which is generally used as an alternative treatment for chemo-resistant NSCLC patients. NSCLC patients with sensitizing EGFR mutation and KRAS mutation were more sensitive to gefitinib [29]. Besides, in the present study, intercellular communication network analysis showed that LNM associated Scissor+ cells dominantly associated with EGF signaling pathway, which possibly provide theoretical basis for the clinical application of gefitinib. Also, the pro-LNM signature based on Scissor^+^ cells showed a favorable predictive ability for LUAD patients with high sensitivity to gefitinib, which may facilitate the individualized therapy.

There are several limitations in this study. First, the pro-LNM gene signature was inferred on the basis of scRNA-seq profiles, further validation of protein expression level in clinical cohorts should be conducted. Besides, the relationship between LNM and the pathophysiological functions of genes in the model needs further investigations, which would facilitate the discovery of new therapeutic targets. Second, although we showed differences in immune microenvironment between LUAD primary tumor and metastatic lymph node, problems such as why B lymphocytes are reducing in the advanced stage of primary tumor but increasing in the metastatic lymph nodes, and whether B cells in lymph nodes particularly affect the prognosis of LUAD patients with lymph node metastasis requires further investigation.

## CONCLUSIONS

This study first revealed the potential genetic drivers for LNM in LUAD based on scRNA-seq data using clonal evolution and transcriptional trajectory analysis. The immune landscape of the tumor microenvironment of LUAD was characterized, and a subset of malignant ECs associated with LNM, named Scissor^+^ cells, was identified. Based on the differentially expressed genes in Scissor^+^ cells, the pro-LNM signature was constructed to predict LNM and was further validated with the use of TCGA LUAD cohort, which exhibited a promising predictive ability to recognize patients with high risk of LNM at an early stage. At last, the potential therapeutic drugs for patients with a high risk of LNM were also predicted, shedding new light on further therapy development for LUAD patients.

## MATERIALS AND METHODS

### Data acquisition

The scRNA-seq profile of the LUAD cohort was obtained from Gene Expression Omnibus (GEO) database (GSE131907) [30]. Data of eleven tumor tissue and ten normal lymph node samples from early stage LUAD patients without prior treatment as well as four tumor tissue and seven metastatic lymph node samples from advanced stage LUAD patients were selected for subsequent analysis. Besides, bulk RNA sequencing data and clinicopathological information of 499 LUAD (167 of them with lymph node metastasis) patients were downloaded from The Cancer Genome Atlas (TCGA, https://portal.gdc.cancer.gov/) database. The study was conducted in accordance with the Declaration of Helsinki (as revised in 2013).

### scRNA-seq data processing

The data from all 32 samples with 116,147 cells were processed and visualized using the Seurat package (4.0.3) in R (4.0.5) [31]. The canonical correlation analysis and mutual nearest neighbors-anchors were used to remove batch effect and integrate scRNA-seq data. Quality control was conducted by excluding cells detecting fewer than 50 genes and cells expressing more than 5% mitochondria-expressing genes. Also, genes detected in less than three cells were filtered. Then, 33,848 cells from primary tumor samples, 37,446 cells from normal lymph node samples, 8,063 cells from advanced stage LUAD samples and 21,479 cells from metastasis lymph node samples were retained for subsequent analysis. To reduce dimensionality, PCA was performed based on variably expressed genes, and the top 20 principle components were further included for cell clustering using t-SNE algorithms. Differential expressed gene (DEG) analysis was performed using the FindAllMarkers function and DEGs were further filtered with an adjusted *P* value < 0.05 and |log_2_(fold change (FC)) |> 0.5. According to various DEG composition patterns, cell clusters were automatically determined and annotated using the SingleR (1.0.1) package, and further manual revisions were performed with reference to the CellMarker database [32]. Canonical cell lineage markers used for cell cluster identification were as follows. B cell: CD19, CD20, CD21, CD23; Endothelial cell: CD31, CD34, CD146, VWF; Epithelial cell: ABCA3, LPCAT1, NAPSA, SFTPB, SFTPC, SLC34A2; Fibroblast: CD36, CD90, CD97, FAP-1, PDGFR-α, PDGFR-β, Vimentin; Macrophage: CD68, CD163, CCL18, CXCL10; Monocyte: CD14, CD36, CD68; NK cell: NKp46, CD56; T cell: CD2, CD3, CD4, CD5, CD8, CXCL13.

### CNV analysis

The large-scale chromosome level CNVs could be predicted based on the gene expression among different samples, which further assisted in inferring malignant cells in tumor. The inferCNV (1.6.0) package was used to calculate the copy number of ECs to distinguish malignant ECs with a cutoff of 0.1. To determine subclonal CNV events and reduce the false positive CNV calls with a threshold of 0.5, hidden Markov models (HMMs) and Bayesian latent mixture models were used [33]. Then, k-means algorithm was applied to hierarchically cluster the CNV scores of all genes for ECs and reference cells (macrophage and monocyte). The ECs with low CNV scores were defined as non-malignant cells, while the ECs with high CNV scores were malignant cells. To illustrate the clonal CNV change, “subcluster” method was further utilized to infer subcluster cells based on CNV value calculated using HMM. Referring to the GRCh38 cytoband information, we converted each CNV to a chromosome arm (p- or q-arm) level change, which was then annotated as a gain or loss [34]. To construct the evolutionary phylogenetic tree, subclones with identical arm level CNVs were collapsed, which was visualized using the UPhyloplot2 algorithm [35].

### Gene enrichment analysis

GSVA was performed to assess the enriched pathways in malignant ECs from primary tumor and lymph node samples [36]. The gene sets were obtained from the Molecular Signatures Database (https://www.gsea-msigdb.org/gsea/msigdb/index.jsp) and CancerSEA Database [37], and the limma package was then applied to performing differential analysis of the enrichment scores of pathways among various cell groups. Pathways with a false discovery rate less than 0.05 and a |*t* value| greater than four were considered significantly differentially enriched.

### SCENIC analysis

The R package SCENIC (https://github.com/aertslab/SCENIC) was further used to analyze the transcriptome factors in various samples [38]. The Gradient Boosting was utilized to infer the co-expression module between transcription factors. The cisTarget Human motif database (https://resources.aertslab.org/cistarget/motif2tf/motifs-v9-nr.hgnc-m0.001-o0.0.tbl) were used for the cis-regulatory motif analysis of each co-expression module. The “aucell” positional argument was applied to score the activity of regulon across single cells. The final result was visualized as a hierarchical clustering heatmap of z-scored enrichment values.

### Scissor algorithm and pseudotime trajectory analysis of maECs

Based on integrated bulk RNA sequencing and scRNA-seq data, Scissor algorithm was applied to identify cell subsets highly related to lymph node metastasis in LUAD [39]. Three input data sources included single-cell expression matrix of maECs, preprocessed LUAD bulk expression matrix from TCGA and LNM-associated phenotype. Then, the data was fitted by a COX regression model with an alpha of 0.05 to select LNM-associated subpopulations. Cells were divided into Scissor^+^ and Scissor^−^ subpopulations according to the symbol of the estimated regression coefficient, which indicated a positive or negative correlation with the target phenotype, respectively.

Monocle (2.18.0) was further applied to analyze pseudotime trajectory based on the transcriptome profiles of Scissor^+^ and Scissor^−^ cells [40]. Genes with a mean expression higher than 0.1 were selected for trajectory analysis, and differentially expressed genes with *q*-value less than 0.01 were further filtered for dimensional reduction using the DDRTree reduction method. According to the gene expression pattern, genes were clustered into subgroups and cells were sorted, trajectory with various branches was constructed according to the expression trend of genes. Branch expression analysis modeling (BEAM) analysis was then performed to identify genes with branch-dependent expression patterns, which might determine the cell fate.

### Cell-cell communication network

CellChat v1.0.0 was applied to quantitatively infer the intercellular communication networks among newly identified Scissor^+^ cells and other cells [41]. Based on the cross-referencing ligand-receptor interaction database (Cell-ChatDB), CellChat incorporated and manually categorized 2,021 molecular interactions into 229 functional-related signaling pathway families. After quantifying the communication probabilities, statistically and biologically significant intercellular communications were identified by contribution degree > 0.25 and *P* < 0.05.

### ssGSEA scores of the bulk RNA sequencing data

SsGSEA was further used to generate a ssGSEA score to estimate the degree of absolute enrichment of certain gene set (pro-LNM signature) in each sample of the TCGA LUAD cohort [42]. The ssGSEA was performed using R package GSVA with default parameters. Then, the relationship between the pro-LNM signature ssGSEA scores and clinicopathological parameters were estimated. Besides, TCGA LUAD cohort was stratified into two groups based on the expression level of pro-LNM signature using the survminer package in R. Kaplan–Meier (K-M) survival analysis was performed to assess the overall survival (OS) of LUAD patients with high and low level of ssGESA scores and compared by the two-sided log-rank test.

### Identification of the drug candidates for LNM patients by targeting Scissor^+^ cells

Expression profile and somatic mutation data of human cancer cell lines (CCLs) were achieved from the Cancer Cell Line Encyclopedia (https://portals.broadinstitute.org/ccle/) [43] and the dependency map (DepMap) portal. Drug sensitivity data of CCLs were downloaded from the Cancer Therapeutics Response Portal (CTRP), which provides values of the area under the dose-response curve to indicate drug sensitivity, and low values of area under the curve (AUC) suggest high sensitivity to treatment. The pRRophetic algorithm was then applied to estimate the chemotherapeutic sensitivity of drugs in the LUAD cohort [44]. Differential drug response analysis was performed by limma package between Scissor^+^ cells and Scissor^−^ cells, and drugs with significant lower AUC (log2FC > 0.01 and *P* < 0.05) were regarded as the candidate drugs for Scissor^+^ cells. Likewise, the drug candidates for patients with LNM in the TCGA cohort were selected by significant lower AUC and a negative correlation between pro-LNM signature and AUC with r < −0.3 and *P* < 0.05.

### Statistical analysis

The statistical analyses were performed using R 4.0.5 software. The independent Student’s *t* test and the χ2 test was applied to compare continuous variables and categorical variables between two groups respectively. We also applied the Mann-Whitney *U* test for categorical variables and nonnormally distributed variables comparison between two groups, and the Kruskal-Wallis test was applied to compare multiple groups. A two-tailed *P* value < 0.05 was considered statistically significant.
